# A Novel Approach to Speed Up Hampel Filter for Outlier Detection

**DOI:** 10.3390/s25113319

**Published:** 2025-05-25

**Authors:** Mario Roos-Hoefgeest Toribio, Alejandro Garnung Menéndez, Sara Roos-Hoefgeest Toribio, Ignacio Álvarez García

**Affiliations:** Department of Electrical, Computer Electronics and Systems Engineering, University of Oviedo, 33003 Oviedo, Spain; marioroos2502@gmail.com (M.R.-H.T.); garnungalejandro@gmail.com (A.G.M.); roostoribiosara.fuo@uniovi.es (S.R.-H.T.)

**Keywords:** signal processing, Hampel filter, outlier detection, real-time filtering, median absolute deviation (MAD)

## Abstract

Outlier detection is a critical task in time series analysis, essential to maintaining data quality and allowing for accurate subsequent analysis. The Hampel filter, a decision filter that replaces outliers in a data window with the median, is widely used for outlier detection in time series due to its simplicity and effectiveness. While effective, its computational complexity, primarily due to the calculation of the Median Absolute Deviation (MAD), poses limitations for large-scale and real-time applications. This paper proposes a novel Hampel filter variant that replaces the MAD with an original estimator (mMAD) that retains statistical robustness but is computationally more efficient. This reduces the filter’s computational complexity from O(N·wlogw) to O(N·w), where *N* is the data length and *w* the window size. The proposed variant significantly lowers processing time and resource consumption, making it especially suitable for large-scale and real-time data processing while preserving robust outlier detection performance.

## 1. Introduction

Detecting outliers [1] is a fundamental aspect of time series analysis, essential for maintaining the accuracy and reliability of data insights. Outliers, defined as data points that significantly deviate from the overall trend, can emerge from various sources, including sensor errors, abrupt environmental changes, or human mistakes in data entry. If not addressed swiftly, these anomalies can distort trends and lead to misleading conclusions, ultimately undermining the validity of analyses and forecasts.

The impact of outlier detection spans multiple fields. In finance, for instance, uncorrected outliers can lead to erroneous predictions regarding stock prices and market volatility, potentially resulting in substantial financial losses [2]. In environmental monitoring, faulty data may generate misleading climate trends, complicating effective policy-making [3]. Healthcare is also affected, as anomalies in patient data can obscure critical health issues or trigger unnecessary alerts [4]. Furthermore, in industrial quality control, ensuring product standards is vital for customer satisfaction and operational efficiency [5]. In manufacturing, real-time monitoring of processes is crucial; if anomalies in sensor readings go undetected, it can lead to equipment failures, increased production costs, or defective products. This underscores the necessity for robust outlier detection methods across various industries to uphold data quality and support informed decision-making.

Various techniques are available for outlier detection, each suited to different data characteristics and application needs [6]. Common methods include statistical approaches, which rely on probabilistic models to identify data points deviating significantly from expected distributions; density-based techniques, which detect anomalies by analyzing the density of surrounding data points, flagging those in sparse regions; clustering methods, which identify outliers as points that do not belong to any dense cluster; and learning-based methods, including active and deep learning, which focus on training models to detect anomalies adaptively [6,7,8].

Among these techniques, the Hampel filter [9] is a well-regarded method in time series data analysis, celebrated for its robustness and simplicity. As a decision-based filter, it replaces central values in a data window with the median if these values are sufficiently far from the median to be deemed outliers. This ability to mitigate the impact of outliers makes the Hampel filter an essential tool in various applications, such as healthcare [10], automotive [11], manufacturing, and industrial measurement [12].

The core strength of the Hampel filter lies in its use of the Median Absolute Deviation (MAD) as a scale estimator, which provides a robust measure of statistical dispersion. This feature enables the Hampel filter to maintain high resistance to the influence of outliers, outperforming many traditional methods that are susceptible to extreme values.

However, a significant limitation of the Hampel filter is its computational complexity. The need to compute the MAD for each moving window in the dataset leads to high processing costs, which becomes a real problem when working with large datasets or applications requiring real-time processing, such as real-time monitoring, anomaly detection in IoT systems, or financial data analysis. In these contexts, the substantial computational expense can significantly hinder its practical usability.

To address this limitation, this paper proposes a modified Hampel filter that approximates the MAD through a secondary application of the median filter on the absolute differences between the original data and its median-filtered version. This modification aims to retain the robust outlier detection capability of the traditional Hampel filter while significantly enhancing its computational efficiency. By reducing the processing time without substantially compromising the effectiveness of outlier detection, the proposed approach offers a promising solution for real-time and large-scale data analysis.

This paper presents a comprehensive analysis of the computational complexity of both the traditional Hampel filter and the proposed variant, accompanied by a formal assessment of the statistical robustness of the new estimator (mMAD), demonstrating robustness levels at least equivalent to those of the Median Absolute Deviation (MAD). To empirically assess the effectiveness of the proposed modification, the accuracy of outlier detection and computational efficiency are evaluated using both synthetic and real-world time series data. The results validate the proposed modified Hampel filter, illustrating a favorable trade-off between computational performance and detection precision, underscoring its potential advantages for large-scale and real-time time series analysis.

The rest of this paper is organized as follows. Section 2 introduces our modified Hampel filter, detailing the approach, robustness, and computational efficiency improvements achieved by approximating the Median Absolute Deviation (MAD) using a secondary median filter. Section 3 presents the experimental setup and compares the performance of the traditional Hampel filter with our proposed variant using both synthetic and real-world time series data. Finally, Section 4 presents the discussion, and Section 5 presents our conclusions.

## 2. Methods

### 2.1. Standard Hampel Filter

The Hampel filter [9] is a robust method for outlier detection that operates on a moving data window. This filter replaces the central value in the window with the median if it is considered an outlier. The process is described as follows.

Let $\left\{x_{t}\right\}$ be a time series. The Hampel filter uses a sliding window of size 2k+1, where *k* represents the number of neighboring points on each side of the central value. For each point $x_{t}$, the following steps are performed.

First, define the sliding window $W_{t}$ centered at $x_{t}$:

(1) $$W_{t}=\left\{x_{t-k},x_{t-k+1},\ldots ,x_{t},\ldots ,x_{t+k}\right\}$$

Next, compute the median of the values within the window:

(2) $$m_{t}=\mathrm{median}\left(W_{t}\right)$$

Then, calculate the Median Absolute Deviation (MAD) within the window. The MAD is a robust measure of statistical dispersion and is defined as

(3) $$S_{t}=1.4826\times \mathrm{median}(|x_{t-i}-m_{t}\left|\right)\;\mathrm{for}\;i=-k,\ldots ,k$$

The factor 1.4826 is used to scale the MAD to be an unbiased estimator of the standard deviation under the assumption of normally distributed data.

To determine if the central value $x_{t}$ is an outlier, check if $x_{t}$ lies outside the interval $\left[m_{t}-tS_{t},m_{t}+tS_{t}\right]$, where *t* is a threshold parameter. The decision rule is given by

(4) $$y_{t}=\left\{\begin{array}{cc} x_{t} & \mathrm{if}\;|x_{t}-m_{t}|\leq tS_{t}\\ m_{t} & \mathrm{if}\;|x_{t}-m_{t}|>tS_{t} \end{array}\right.$$

The robustness of the Hampel filter comes from the use of the median and MAD, which are less affected by outliers compared to the mean and standard deviation. This makes the Hampel filter particularly effective in maintaining the integrity of the data while identifying and mitigating the impact of outliers.

### 2.2. Proposed Hampel Filter with Modified MAD (mMAD)

The proposed modified Hampel filter is an adaptation of the standard Hampel filter that aims to improve computational efficiency by approximating the Median Absolute Deviation (MAD) using a secondary median filter. This method reduces computational complexity while maintaining robustness in outlier detection. The process is described as follows.

Let $\left\{x_{t}\right\}$ be a time series. The modified Hampel filter proposed uses a sliding window of size 2k+1, where *k* represents the number of neighboring points on each side of the central value. For each point $x_{t}$, the following steps are performed.

First, define the sliding window $W_{t}$ centered at $x_{t}$:

(5) $$W_{t}=\left\{x_{t-k},x_{t-k+1},\ldots ,x_{t},\ldots ,x_{t+k}\right\}$$

Next, compute the median of the values within the window:

(6) $$m_{t}=\mathrm{median}\left(W_{t}\right)$$

Then, calculate the absolute deviations between each point in the window and their corresponding medians. For each point within the window, compute

(7) $$d_{t-i}=\left|x_{t-i}-m_{t-i}\right|,\;\mathrm{for}\;i=-k,\ldots ,k$$

Here, $m_{t-i}$ is the median computed for the window centered at $x_{t-i}$.

Compute the mMAD by applying a median filter to the absolute deviations $d_{t-i}$ within a window of size $2k^{\prime }+1$, where $k^{\prime }$ is the number of neighboring deviations on each side:

(8) $$S_{t}=\mathrm{median}\left(D_{t}\right),\;\mathrm{where}\;D_{t}=\left\{d_{t-k^{\prime }},d_{t-k^{\prime }+1},\ldots ,d_{t},\ldots ,d_{t+k^{\prime }}\right\}$$

To determine if the central value $x_{t}$ is an outlier, check if $x_{t}$ lies outside the interval $\left[m_{t}-tS_{t},\;m_{t}+tS_{t}\right]$, where *t* is a threshold parameter. The decision rule is given by

(9) $$y_{t}=\left\{\begin{array}{cc} x_{t}, & \mathrm{if}\;|x_{t}-m_{t}|\leq tS_{t}\\ m_{t}, & \mathrm{if}\;|x_{t}-m_{t}|>tS_{t} \end{array}\right.$$

The key difference in this modified method is the approximation of the MAD through median filtering of the absolute deviations, which reduces computational complexity.

The robustness of the modified Hampel filter is retained through the use of medians in both the central tendency and dispersion measures, which are less affected by outliers compared to means and standard deviations. This approach effectively identifies and mitigates the impact of outliers while enhancing computational efficiency.

### 2.3. Analysis of the Robustness of the Proposed Estimator

A fundamental property of any scale estimator is its robustness, which ensures reliability in the presence of outliers or contaminated data. To establish the robustness of the modified MAD (mMAD), this section presents a comprehensive analysis from three perspectives. First, the statistical stability of the mMAD is examined by comparing its deviation from the classical MAD under controlled perturbations. Then, the breakdown point is analyzed to determine the maximum fraction of contaminated observations that the mMAD can tolerate while maintaining a reliable estimate. Finally, the influence function is studied to assess the estimator’s sensitivity to infinitesimal contamination. These analyses demonstrate that the mMAD retains at least the same robustness as the classical MAD.

#### 2.3.1. Robustness of the Proposed Estimator

This section aims to demonstrate the robustness of the mMAD, which is the principal objective of the proposed estimator presented in this article. This is stated formally as follows:

**Claim** **1.** *The MAD approximation based on local medians is at least as robust as, and potentially more robust than, the original MAD computed using a global median.*

The classical MAD is defined as

(10) $$\sigma_{\mathrm{MAD}}=\mathrm{med}\left(\left|x_{i}-\mathrm{med}\left(x\right)\right|\right),$$

where med(x) represents the global median of the dataset. The modified MAD (mMAD) replaces this global median with local medians. Given a window $W_{i}$ of size 2k+1, the local median is defined as

(11) $${\tilde{\mu }}_{i}=\mathrm{med}\left(W_{i}\right),$$

leading to the modified estimator

(12) $$\sigma_{\mathrm{mMAD}}=\mathrm{med}\left\{\left|x_{j}-{\tilde{\mu }}_{j}\right|:x_{j}\in W_{i}\right\}.$$

The robustness of the mMAD is established through two fundamental properties of the median:

**Axiom** **1** (reverse triangle inequality)**.** *For any real numbers x and y,*

(13) $$\left||x|-|y|\right|\leq \left|x-y\right|.$$

*This inequality implies that the absolute deviation of a value cannot change more than the difference between two reference points.*

**Axiom** **2** (Lipschitz continuity of the median)**.** *A function f is L-Lipschitz [13] if there exists a constant L such that for any two inputs A,B,*

(14) |f(A)−f(B)|≤L·∥A−B∥,

*where ∥A−B∥ represents the distance between the inputs. The median is 1-Lipschitz under the infinity norm, implying that if all elements in a dataset change by at most δ, then the median also changes by at most δ:*

(15) |med(A)−med(B)|≤δ.

*This property ensures that small perturbations in the data produce at most proportionally small changes in the median.*

To demonstrate that the mMAD retains the robustness of the classical MAD, the deviation between both estimators is analyzed. The global and local MAD are defined, respectively, as

(16) $$\sigma^{*}=\mathrm{med}\left\{|x_{j}-m|\right\},\;{\tilde{\sigma }}_{i}=\mathrm{med}\left\{|x_{j}-{\tilde{m}}_{j}|\right\},$$

where *m* denotes the global median and ${\tilde{m}}_{j}$ represents the local median in window $W_{i}$.

By applying the reverse triangle inequality,

(17) $$\left|\;|x_{j}-m|-|x_{j}-{\tilde{m}}_{j}|\;\right|\leq \left|m-{\tilde{m}}_{j}\right|.$$

Assuming that the local median remains within δ of the global median,

(18) $$|m-{\tilde{m}}_{j}|\leq \delta \;\forall j\in W_{i},$$

the sequences

(19) $$A_{j}=|x_{j}-m|,\;B_{j}=\left|x_{j}-{\tilde{m}}_{j}\right|$$

satisfy the bound

(20) $$|A_{j}-B_{j}|\leq \delta .$$

By the Lipschitz continuity of the median,

(21) $$\left|\mathrm{med}(A)-\mathrm{med}(B)\right|\leq \delta ,$$

which leads to the conclusion

(22) $$|\sigma^{*}-{\tilde{\sigma }}_{i}|\leq \delta .$$

This result establishes that when the local median deviates from the global median by at most δ, the local MAD remains within δ of the global MAD. Consequently, the mMAD preserves at least the same robustness as the classical MAD.

If outliers are uniformly distributed, both estimators exhibit equivalent robustness. However, in scenarios where outliers are clustered in specific regions, the local MAD demonstrates improved performance by remaining unaffected in non-contaminated windows.

Since the median is a stable operator under bounded perturbations, the mMAD retains the robustness of the classical MAD while providing significant computational advantages.

#### 2.3.2. Breakdown Point Analysis

The breakdown point of an estimator is the largest fraction ϵ of contaminated data it can tolerate before its estimate becomes arbitrarily large.

Let *F* be the true underlying distribution of the data, and let *G* be a contaminated distribution, where some observations have been replaced by arbitrary values [14]. Define $G_{\epsilon }$ as the set of all possible distributions obtained by replacing at most an ϵ-fraction of the observations in *F* with arbitrary values.

For any contamination fraction ϵ<0.5, both the Median Absolute Deviation (MAD) and the modified Median Absolute Deviation (mMAD) satisfy

(23) $$\underset{G\in G_{\epsilon }}{\sup}\left|\sigma \left(F\right)-\sigma \left(G\right)\right|<\infty .$$

meaning that neither $\sigma_{\mathrm{MAD}}$ nor $\sigma_{\mathrm{mMAD}}$ diverges under such contamination, ensuring that their breakdown point is 50%.

Since for any $G\in G_{\epsilon }$ with ϵ<0.5, the deviation between scale estimators |σ(F)−σ(G)| remains finite, the result follows.

- Classical MAD: Since the median has a BP of 50%, the MAD inherits this property [14]. Contamination of up to 50% of the data cannot arbitrarily perturb the median of absolute deviations.
- Modified MAD (mMAD): For the mMAD, let $W_{i}$ be a sliding window of size 2k+1 centered at $x_{i}$. The local median ${\tilde{\mu }}_{i}=\mathrm{med}\left(W_{i}\right)$ has a BP of 50% within $W_{i}$. Even if ϵ<0.5 of the entire dataset is contaminated, each window $W_{i}$ contains at most ϵ(2k+1) outliers. Since ϵ<0.5, we have
  ϵ(2k+1)<k+0.5,
  ensuring the local median ${\tilde{\mu }}_{i}$ remains bounded. The absolute deviations $|x_{i}-{\tilde{\mu }}_{i}|$ are then filtered by another median (mMAD), which also has a BP of 50%.

Thus, $\sigma_{\mathrm{mMAD}}$ inherits the 50% contamination tolerance threshold. In practical terms, as long as no more than half of the observations in any sliding window are contaminated, whether by isolated spikes or consecutive pulses, the MAD and mMAD remain bounded and robust. Only once the proportion of outliers exceeds 50% (e.g., 6 of 11 samples) is the scale estimate potentially compromised. This identical threshold for both the traditional and proposed filters ensures equivalent resilience to closely spaced anomalies.

#### 2.3.3. Influence Function Analysis

The influence function (IF) [15] quantifies how small perturbations in the data affect an estimator. Formally, it is defined as

(24) $$\mathrm{IF}\left(x;T,F\right)=\lim_{\epsilon \to 0}\frac{T(\left(1-\epsilon \right)F+\epsilon \Delta_{x})-T\left(F\right)}{\epsilon }.$$

This function provides insight into the robustness of an estimator by measuring its sensitivity to outliers. A robust estimator is characterized by a bounded influence function, ensuring that extreme values do not unduly affect the final estimate [14].

For the median, the influence function is given by

(25) $$\mathrm{IF}\left(x;\mathrm{med},F\right)=\frac{1}{2f(m)}\mathrm{sign}\left(x-m\right),$$

where f(m) is the density of the distribution at the median. Since this function is bounded, the median is robust against outliers. The classical MAD, which is derived from the median, inherits this robustness and maintains an influence function that does not grow unbounded, ensuring that small perturbations in the data do not cause significant changes in the estimate. This property can be summarized as

(26) $$\mathrm{IF}\left(x;\sigma_{\mathrm{MAD}},F\right)=O\left(1\right),$$

indicating that the effect of an infinitesimal contamination remains controlled.

The modified MAD (mMAD), which relies on local medians instead of a global median, follows a similar principle. The robustness of the mMAD depends on the behavior of these local medians, which vary smoothly under small perturbations. By considering the effect of a single perturbed observation, the influence function of the local median satisfies

(27) $$\mathrm{IF}\left(x;{\tilde{\mu }}_{i},F\right)\leq \frac{1}{2f({\tilde{\mu }}_{i})},$$

where $f({\tilde{\mu }}_{i})$ represents the density function evaluated at the local median. Since each local median is stable, the absolute deviations $|x_{i}-{\tilde{\mu }}_{i}|$ inherit this boundedness.

Ultimately, the mMAD estimator applies another median operation over these absolute deviations. The result is an influence function bounded by

(28) $$\mathrm{IF}\left(x;\sigma_{\mathrm{mMAD}},F\right)\leq \max_{i\in W_{i}}\mathrm{IF}\left(x;{\tilde{\mu }}_{i},F\right)+L\delta ,$$

where *L* is a Lipschitz constant that controls how much local medians can change under small perturbations, and δ is the maximum deviation induced by contamination. Since both terms are bounded, the influence function of the mMAD remains controlled, confirming that it retains the robustness of the classical MAD.

### 2.4. Computational Complexity Comparison

The Hampel filter is a technique used to identify and replace outliers in a data series employing a sliding window approach. In this subsection, we detail how the Hampel filter operates and analyze the computational complexity ([16], Chapter 3) of both the original Hampel filter and a computationally efficient variant.

#### 2.4.1. Original Hampel Filter

The original Hampel filter consists of the following steps:

1. Median filter calculation:
  - A median filter is applied to the original data series to obtain $m_{i}$.
  - Complexity: O(N·w), since the median filter operates over a sliding window [17].
2. Calculation of absolute deviations from the median:
  - For each point, the absolute deviation from the median within the window is calculated as
    (29) $$D_{i}=\left|x_{i}-m_{i}\right|$$
  - Complexity: O(N), as it involves an elementary operation on each data point.
3. Calculation of the Median Absolute Deviation (MAD):
  - The median of the absolute deviations within each window is computed as
    (30) $${\mathrm{MAD}}_{i}=\mathrm{median}\left(D_{i-h},\ldots ,D_{i+h}\right)$$
  - Complexity: O(N·wlogw), since each window needs to be sorted to find the median.
4. Determination of the outlier threshold:
  - A threshold is computed by multiplying the MAD by a scaling factor κ, typically defined as
    (31) $$T_{i}=\kappa \cdot {\mathrm{MAD}}_{i}$$
  - Complexity: O(N), as it involves a simple multiplication for each point.
5. Replacement of outliers:
  - If the absolute deviation of a point exceeds the threshold, i.e., $D_{i}>T_{i}$, the point is considered an outlier and is replaced with the median $m_{i}$ of its window.
  - Complexity: O(N), involving a comparison and possible substitution for each data point.

The most computationally intensive part of the algorithm is the calculation of the median within each window, which has a complexity of O(N·wlogw). Therefore, the total computational complexity of the original Hampel filter is O(N·wlogw).

#### 2.4.2. Proposed Hampel Filter Variant

The proposed alternative variant of the Hampel filter reduces computational complexity by modifying the calculation of the MAD. The steps are as follows:

1. Median filter calculation:
  - A median filter is applied to the original data series to obtain $m_{i}$.
  - Complexity: O(N·w), since the median filter operates over a sliding window.
2. Calculation of absolute deviations from the initial median:
  - For each point, the absolute deviation from the median is calculated:
    (32) $$D_{i}=\left|x_{i}-m_{i}\right|$$
  - Complexity: O(N), involving an element-wise operation.
3. Application of median filter to absolute deviations (MAD):
  - A median filter is applied to the absolute deviations $D_{i}$ to obtain ${\mathrm{MAD}}_{i}$.
  - Complexity: O(N·w), as it involves another median filter over a sliding window.
4. Calculation of the outlier threshold:
  - The threshold for each point is computed as
    (33) $$t_{i}=\kappa \cdot {\mathrm{MAD}}_{i}$$
  - Complexity: O(N).
5. Application of the Hampel filter:
  - For each point, if $D_{i}>T_{i}$, the point is considered an outlier and is replaced with the median $m_{i}$.
  - Complexity: O(N).

The major computational cost in this variant arises from applying the median filter twice, but it avoids sorting the absolute deviations in each window to compute the MAD. The total computational complexity is O(N·w), where *N* is the number of data points and *w* is the window size.

#### 2.4.3. Comparison

The proposed Hampel filter variant significantly improves computational efficiency by eliminating the need for sorting within each sliding window to calculate the MAD. This adjustment removes the logarithmic term present in the original filter’s complexity, which is a major contributor to its higher computational cost, especially when applied to large datasets or real-time data streams. As a result, the variant reduces both processing time and resource consumption:

- Original Hampel filter: O(N·wlogw).
- Hampel filter variant: O(N·w).

**Figure 1:**
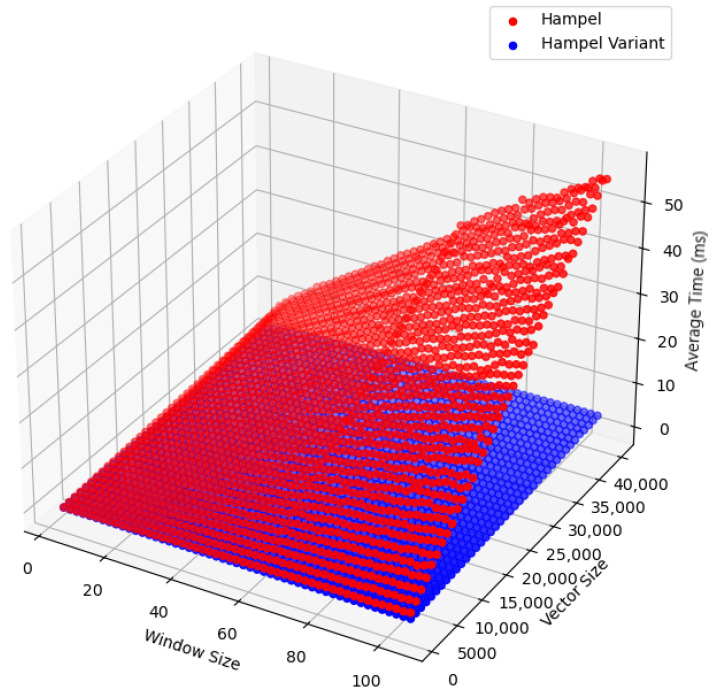
Comparison of computational complexity: original Hampel filter vs. modified variant.

The performance comparison is shown in Figure 1. The 3D plot illustrates the average processing time as a function of both the window size and vector size. As depicted, the original Hampel filter (red) experiences a steep increase in processing time as the window size and vector size grow due to the need for sorting within each sliding window to compute the MAD. In contrast, the modified Hampel filter (blue) exhibits a much more gradual rise in processing time thanks to the elimination of the sorting step.

## 3. Experiments and Result

This section evaluates the performance of the Hampel filter and our proposed computationally efficient variant through two complementary experiments, following the methodology outlined in [18]. The experiments were conducted on a workstation equipped with an AMD Ryzen 9 4900HS CPU (AMD, Santa Clara, CA, USA), 16 GB RAM, and running Ubuntu 22.04 LTS. No GPU acceleration was required for the reported results.

The first experiment analyzes their effectiveness using synthetic data, designed to include various signal components and noise, allowing a precise assessment of their ability to recover signals in the presence of impulsive noise. In the second experiment, both filters are applied to real-world data, specifically with the “gipi” sequence of Italian industrial production from 1981 to 1996 [19], to evaluate their performance in detecting and correcting seasonal outliers.

### 3.1. Evaluating Hampel Filter Performance with Synthetic Data

To quantitatively evaluate the performance of the Hampel filter, we construct a 520-point synthetic sequence that combines multiple signal components and noise types. First, a step-and-ramp backbone is defined over all 520 samples: it starts at 0 for k=1 to k=40, rises linearly to 1 by k=100, remains at 1 until k=140, climbs to 2 by k=220, then descends back to 0 by k=300, dips to −1 from k=320 to k=400, and finally ramps up linearly from 0 to 1 over the last 100 points (k=421 to k=520).

Next, low-level Gaussian noise with μ=0 and σ=0.1 is added only to the first 240 samples, emulating small-scale measurement error. A sinusoidal component of amplitude 0.3 and period 29 is then superimposed on the interval k=101 through k=420, introducing smooth periodic fluctuations.

**Figure 2:**
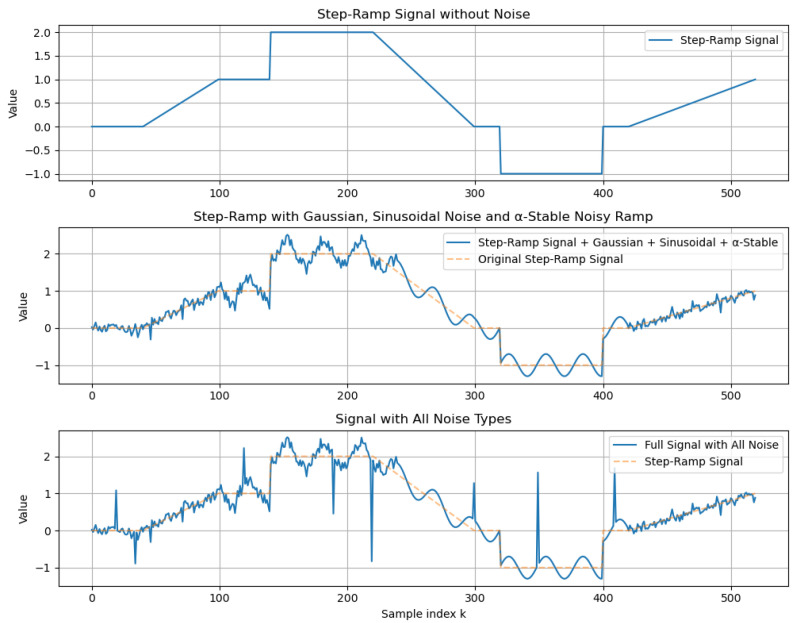
Simulated signal combining step–ramp, sinusoidal components, Gaussian noise, and impulsive outliers.

Finally, over the last 100 points (k=421–520), the linear 0–1 ramp is perturbed by α-stable noise (α=1.7, β=0, scale = 0.1) [20], generating occasional large deviations that stress-test the filter’s ability to reject extreme impulses. The resulting “full” signal thus includes a clean piecewise ramp, local Gaussian and sinusoidal variations, scattered impulsive spikes, and a heavy-tailed noisy ramp segment (see Figure 2).

To simulate impulsive outliers, eight isolated spikes are injected at specific base indices: k=20 (+1), k=35 (−1), k=120 (+1), k=190 (−1.5), k=220 (−2.5), k=300 (+1), k=350 (+2.5), and k=410 (+1.5).

The primary objective is to assess the effectiveness of the Hampel filter in removing isolated spikes while preserving low-level details, particularly the sinusoidal component. Given that the Hampel filter and its variants are more suited to removing impulsive noise rather than smoothing low-level noise, the focus will be on their performance in eliminating impulsive noise.

The filter’s performance is evaluated using two metrics: Root Mean Square Error (RMSE) and Mean Absolute Error (MAE). These metrics provide a quantitative measure of how well the filter performs in different scenarios.

The evaluation is conducted across four qualitatively distinct subsequences. The first segment, “Noise Only” (k=1 to k=100), consists of the step-and-ramp backbone contaminated with low-level Gaussian noise (σ=0.1) and the eight fixed impulsive spikes. The second segment, “Noise + Sine” (k=101 to k=240), adds the sinusoidal fluctuation (amplitude 0.3, period 29) on top of the same noise components. The third segment, “Sine Only” (k=241 to k=420), features the ramp plus sinusoid and isolated spikes, with Gaussian noise removed. Finally, the fourth segment, “Heavy-Tailed Ramp” (k=421 to k=520), is a linear 0→1 ramp perturbed by α-stable noise (α=1.7,β=0,scale=0.05), introducing rare but large impulses.

**Figure 3:**
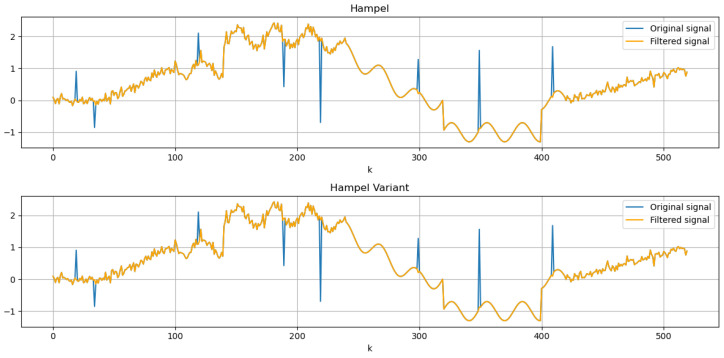
Step–ram signal filtered with the traditional and new versions of the Hampel filter (window size = 11, t=2).

For the evaluation of the filter, a signal-recovery problem is considered: the removal of impulsive noise to restore a composite signal consisting of a step-and-ramp sequence perturbed by additive Gaussian noise, a sinusoidal component, and α-stable noise. The filtering results obtained with both the standard Hampel filter and its proposed variant are shown in Figure 3.

**Figure 4:**
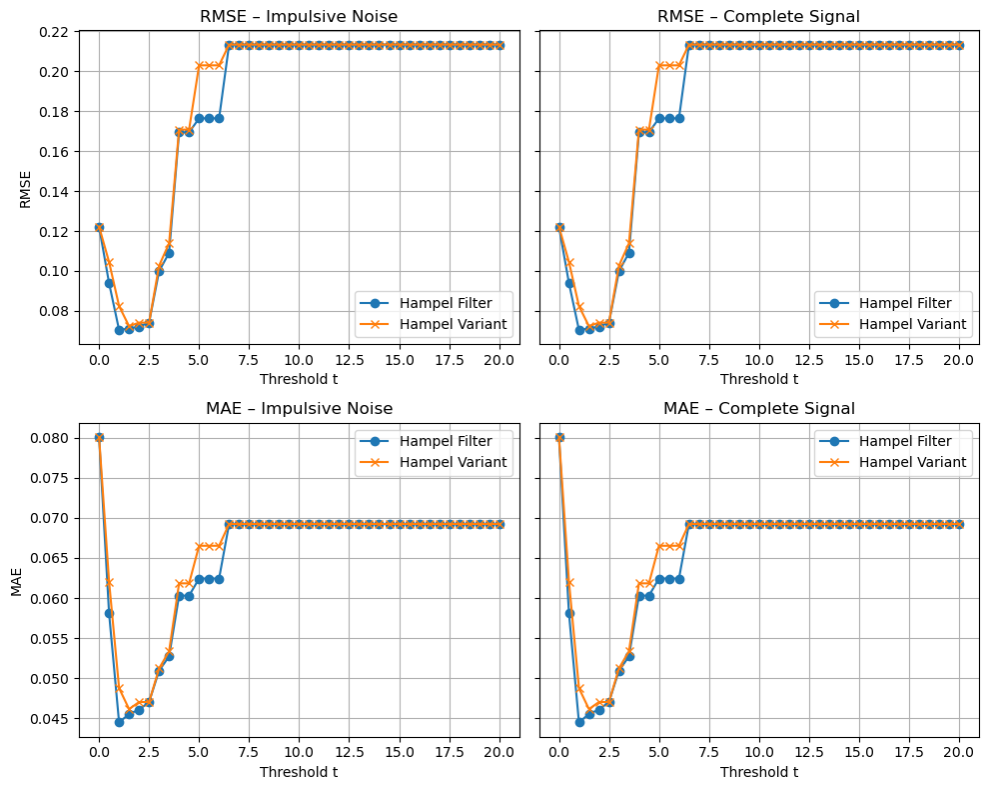
RMSE and MAE of the filtered signal versus *t* for impulsive and complete signals, using a window size of 11.

In the upper diagram, the traditional Hampel filter is applied to the impulsive-noise scenario, whereas the lower diagram shows the proposed variant operating on the same signal. Both plots use a window size of 11 samples and a threshold t=2, values that were found experimentally to be optimal for the point density in these tests: a length-11 window strikes a balance between smoothing and retention of local features, and t=2 effectively discriminates outliers without removing genuine signal variations, as can be seen in Figure 4.

By employing RMSE and MAE, two commonly used metrics for assessing the accuracy of signal reconstruction, we gain deeper insights into the balance between noise suppression and signal preservation achieved by the filtering strategies. Formally, the Root Mean Squared Error (RMSE) is defined as

(34) $$\mathrm{RMSE}=\sqrt{\frac{1}{N}\sum_{k=1}^{N}{\left({\hat{y}}_{k}-y_{k}\right)}^{2}}$$

where $y_{k}$ represents the true signal value at time *k*, ${\hat{y}}_{k}$ the estimated signal value after filtering, and *N* the total number of samples. The RMSE places a higher penalty on large deviations from the ground truth, making it particularly sensitive to impulsive spikes that remain after filtering.

On the other hand, the Mean Absolute Error (MAE) is given by

(35) $$\mathrm{MAE}=\frac{1}{N}\sum_{k=1}^{N}\left|{\hat{y}}_{k}-y_{k}\right|$$

Unlike RMSE, the MAE treats all errors uniformly, providing a measure of the average deviation that is less influenced by outliers. By considering both metrics simultaneously, it is possible to identify situations in which a given filter might excel at reducing large errors (reflected in a lower RMSE) but may still leave a significant number of smaller deviations that raise the MAE or vice versa.

Figure 4 shows that the filtered signals produced by both methods overlap perfectly, showing that the Hampel filter variant achieves similar levels of noise suppression and signal preservation as the standard implementation. This highlights that the filters are functionally equivalent in their ability to clean the signal while maintaining its features.

The results demonstrate that the performances of the Hampel filter and its variant are remarkably similar across all tested values of the threshold parameter *t*. As Figure 4 shows, both filters achieve equivalent RMSE and MAE values, indicating that neither approach offers a significant advantage in terms of noise suppression or signal preservation.

Moreover, as we stated earlier, the plot reveals that both metrics exhibit a minimum around t=2, which reflects an optimal balance between aggressive filtering and retention of signal detail. Lower values of *t* tend to remove genuine features along with noise, while higher values may fail to eliminate all outliers.

### 3.2. Real Data Example

To evaluate the performance of the Hampel filter and the proposed Hampel filter variant on real-world data, both methods were applied to a publicly available time series dataset. Specifically, the “gipi” sequence from the “tsoutliers” R package was used [21], which represents a monthly time series of the Italian industrial production index from 1981 to 1996, consisting of 192 observations. These experiments follow the methodology outlined in [18].

This dataset contains significant outliers occurring at regular 12-month intervals, known as seasonal outliers. These anomalies can distort data analysis if not properly addressed, making it essential to filter them out for accurate interpretation of the time series.

In this experiment, both the original Hampel filter and the proposed variant were applied to the dataset using a window size of w=5 and a threshold value of t=2. The window size determines the number of neighboring data points considered, while *t* defines the sensitivity of the filter to deviations from the median.

**Figure 5:**
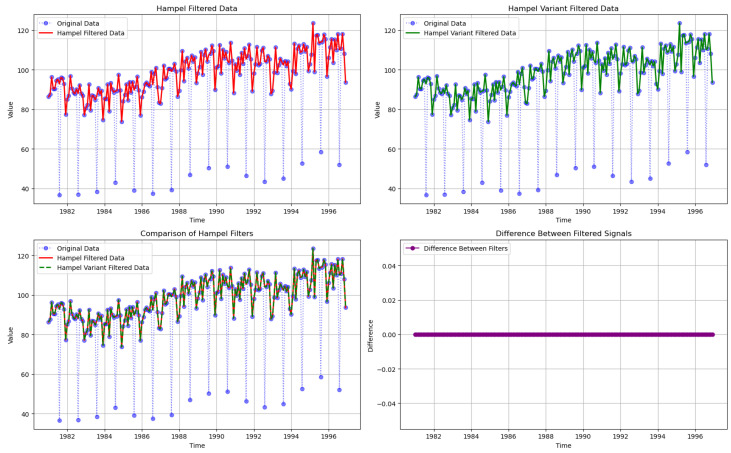
Results of applying the Hampel filter and its variant to the time series.

After filtering, the results were compared by analyzing the filtered time series and examining the differences between the two methods, see Figure 5. Both filters effectively identified and corrected the outliers in the dataset, significantly reducing the impact of the seasonal anomalies. The essential characteristics and trends of the time series were preserved in both cases.

Overall, both the original and variant Hampel filters effectively reduce the seasonal outliers, preserving the main characteristics of the time series. While both approaches yield similar results, the variant offers improved computational efficiency, making it a practical choice for larger datasets or real-time applications.

## 4. Discussion

In this paper, we introduced a computationally efficient variant of the Hampel filter that approximates the Median Absolute Deviation (MAD) using a secondary median filter. Our results demonstrate that this modification achieves equivalent performance to the original Hampel filter while significantly reducing computational complexity. This enhancement is particularly useful in real-time or large-scale applications where computational resources and processing speed are critical factors. Despite the reduced computational cost, the variant maintains the robustness of the original Hampel filter in detecting outliers and recovering signals in the presence of noise, as shown in both synthetic and real-world datasets.

The proposed modified Hampel filter (mMAD) offers a key advantage in handling time series data with impulsive noise and seasonal outliers. Our experiments demonstrate that the variant performs similarly to the original Hampel filter when applied to both synthetic data and the “gipi” dataset [19]. This performance is achieved with lower computational cost, making the variant a practical choice for large-scale or real-time data applications. By using median-based computations for both central tendency and dispersion, the filter remains robust to extreme values, ensuring accurate outlier detection.

Additionally, the threshold parameter *t* plays a critical role in the filter’s sensitivity. While scaling factors are essential in theoretical models, our results highlight that the threshold is more influential in practice. The ability to fine-tune this parameter allows the Hampel filter to adapt to different data characteristics, making it versatile across diverse real-world scenarios.

In conclusion, the modified Hampel filter proposed provides a computationally efficient alternative to the original, maintaining robustness while offering substantial reductions in processing time. The choice of threshold *t* remains crucial for the filter’s effectiveness, and further research could explore dynamic adjustments to this parameter based on real-time data.

Future work will focus on extending the Hampel filter variant to higher-dimensional data, exploring its application and performance in signal processing tasks involving 2D and 3D datasets. This includes assessing its effectiveness in filtering outliers in images and volumetric data, which are common in fields such as computer vision and industry applications. Studying the filter’s performance in these contexts will provide deeper insights into its potential for broader applications.

## 5. Conclusions

The experiments conducted demonstrate that both the original Hampel filter and its computationally efficient variant proposed in this article are effective in identifying and correcting outliers in time series data. Both methods preserve the essential characteristics and trends of the dataset while significantly reducing the impact of anomalies. This ensures the reliability of subsequent data analyses and modeling.

A key distinction between the two filters lies in their computational efficiency. The variant reduces the algorithmic complexity from O(N·wlogw) to O(N·w), resulting in faster processing times without compromising filtering performance.

In summary, while both filters offer equivalent performance in terms of outlier detection and correction, the Hampel filter variant provides substantial benefits in computational efficiency. This efficiency gain makes it a preferable choice for modern data processing tasks where computational resources and speed are critical factors.

## Data Availability

The data presented in this study are available from the corresponding author on request.
